# Treatment patterns and associated outcomes among patients with HER2+ metastatic breast cancer in the United States: an observational cohort study

**DOI:** 10.1093/oncolo/oyae280

**Published:** 2025-04-07

**Authors:** Clara Lam, Della Varghese, Jenna Collins, Beth Nordstrom, Brian Murphy, Sandhya Mehta, Eleanor Faherty

**Affiliations:** AstraZeneca Pharmaceuticals, LP, Gaithersburg, MD, United States; AstraZeneca Pharmaceuticals, LP, Gaithersburg, MD, United States; Evidera, Waltham, MA, United States; Evidera, Waltham, MA, United States; Evidera, Waltham, MA, United States; Daiichi Sankyo, Inc., Basking Ridge, NJ, United States; Daiichi Sankyo, Inc., Basking Ridge, NJ, United States

**Keywords:** human epidermal growth factor receptor 2, metastatic breast cancer, survival, treatment patterns

## Abstract

**Background:**

With treatment options for human epidermal growth factor receptor 2-positive (HER2+) metastatic breast cancer (mBC) expanding, updated assessments of contemporary treatment patterns and clinical outcomes are needed. This study aimed to conduct such an assessment using data from real-world oncology practices.

**Materials and methods:**

Adult HER2+ mBC patients initiating first-line (1L) treatment from January 2013 to January 2021 (index date) were selected from the US Flatiron Health database and followed through January 2022. Patient characteristics and treatment patterns were summarized. Clinical outcomes were examined using Kaplan-Meier analyses.

**Results:**

Among 2074 HER2+ mBC patients with at least 1 line of therapy (LoT), median age was 61 years and 62.8% had known hormone receptor-positive disease. During a median follow-up of 26.0 months, 1159 (55.8%) had at least 2 LoTs, and 584 (28.2%) had 3 or more. The most common 1L regimens included docetaxel, trastuzumab, and pertuzumab (THP; 38.9%) followed by THP+ platinum agent (7.5%) and ado-trastuzumab emtansine (T-DM1) monotherapy (6.1%). By the end of follow up, 18.1% of patients remained on treatment, 20.2% died, and 5.8% discontinued without starting a new treatment. Median overall survival from 1L start was 40.3 months.

**Conclusions:**

Approximately one-quarter of the patients died or discontinued 1L therapy without receiving further treatment. Overall survival from the start of 1L was just over 3 years. This highlights a need for more effective therapies in earlier LoTs that prolong the time to progression and provide longer clinical benefits.

## Teaser

With the expanding treatment options for HER2+ metastatic breast cancer, updated assessments of contemporary treatment patterns and clinical outcomes are needed. This study conducted such an assessment using data from real-world oncology practices.

**Implications for practice**

There are several drugs available to treat HER2+ mBC, including some that are more recently approved. This study sought to better understand how these drugs are used and how well they work in patients with HER2+ mBC receiving treatment in largely community-based clinics.

Treatment patterns varied, and more than half of the patients moved to a new therapy within approximately 1 year of their first treatment suggesting rapid progression of disease. The findings indicate that these patients need more effective first treatments that can prevent cancer from growing, spreading, or worsening and provide longer benefits.

## Introduction

Breast cancer (BC) is the most common malignancy diagnosed in women, representing nearly 15% of all new cases of cancer in the United States.^1^ For 2023, a total of 297 790 new cases of invasive BC and 43 170 associated deaths were predicted.^1,2^ In 2020, approximately 168 000 women in the United States had metastatic BC (mBC),^3,4^ 75% of whom were initially diagnosed with stage I-III BC and later progressed to mBC. Approximately 6% of women with BC present with mBC at the initial diagnosis.^4^ The 5-year survival rate for women with mBC is estimated at 31%.^1^

BC is categorized into distinct molecular subtypes based on the presence of hormone receptors (HR) and human epidermal growth factor receptor 2 (HER2).^5^ HER2-positive (HER2+) mBC is an aggressive subtype that accounts for approximately 20% of all BC cases,^6^ and patients with this subtype may show an increased likelihood of developing metastasis. For instance, approximately 25%-50% of HER2+ mBC patients will develop brain metastases.^7^ Although mBC remains incurable, its prognosis has significantly improved during the past several years with the introduction of targeted therapies. A retrospective cohort study reported median overall survival (mOS) of 39 months, 58 months, and “not reached” in the years 2008, 2013, and 2014 onward, respectively, in patients with the HER2+ subtype.^8^

Currently, pertuzumab and trastuzumab combined with a taxane, commonly referred to as THP, are the standard first-line (1L) treatment options for HER2+ mBC. Trastuzumab emtansine (T-DM1) was considered the standard of care (SOC) in the second line of treatment (LoT; 2L) during the study period.^9^

In December 2019, February 2020, and April 2020, respectively, trastuzumab deruxtecan (T-DXd), neratinib, and tucatinib were approved for patients who had previously received anti-HER2-based regimens.^10-12^ T-DXd was granted US Food and Drug Administration approval based on the results from a phase II clinical trial of 184 women with HER2+ mBC who had received previous treatment.^13^ These results showed that patients treated with T-DXd had an overall response rate of almost 70% and median progression-free survival (mPFS) of 19.4 months.^14^ Neratinib was approved in combination with capecitabine for patients who had received 2 or more prior anti-HER2-based regimens in the metastatic setting. The efficacy and safety of this combination were assessed in the NALA trial and showed longer overall survival (OS) (21 vs 18.7 months; not significant) and mPFS (5.6 vs 5.5 months) than in patients receiving lapatinib plus capecitabine.^12^ Tucatinib was approved based on the results of a phase III, double-blind, placebo-controlled clinical trial in which patients with HER2+ mBC received either tucatinib or placebo with trastuzumab and capecitabine. Compared with those who received the placebo, patients who received tucatinib showed significantly increased PFS (median duration: 7.8 months vs 5.6 months) and OS (median duration: 21.9 months vs 17.4 months).^15^ Subsequently, the DESTINY-Breast03 trial found PFS to be 28.8 months in patients treated with T-DXd compared with 6.8 months in those treated with T-DM1 (hazard ratio: 0.33 [95% CI, 0.26-0.43]).^16^ The percentage of patients who were alive at 12 months was 94.1% and 85.9% for those treated with T-DXd and T-DM1, respectively (hazard ratio: 0.55 [95% CI, 0.36-0.86]).^17^

Limited real-world data are available regarding the current treatment patterns and associated clinical outcomes, considering the advent of these recently approved therapeutic options for patients with HER2+ mBC. Accordingly, the objective of this study was to describe the demographic and clinical characteristics and the treatment patterns and to summarize clinical outcomes by treatments received after a metastatic diagnosis among patients with HER2+ mBC, overall and further stratified by HR status.

## Materials and methods

### Data source

The Flatiron Health Data Repository includes comprehensive information on patients with cancer derived from electronic health records (EHR). It comprises patient-level data that are structured (eg, data points organized in a predefined manner, such as dropdown fields) and unstructured (eg, free text from a physician’s note or a scanned pathology report), curated via technology-enabled abstraction. The data include processed longitudinal patient-level EHR data, such as demographics, deep diagnosis information (eg, staging, histopathology, and biomarkers), treatments, and outcomes (eg, mortality). The database is built on the Flatiron Health Provider Network of more than 265 cancer clinics (largely community-based), representing more than 880 sites of care including more than 2 million patients with cancer in the United States, across all tumor types, available for analysis. These patients are broadly distributed across the country. A limited number of National Comprehensive Cancer Network (NCCN) member institutions also participate in the Flatiron Health data collection.

### Study population

The full study cohort included patients aged ≥18 years who had histologically or cytologically documented BC with evidence of metastatic disease, and receipt of systemic anti-cancer therapy up to 14 days before, on, or after mBC diagnosis date and between January 1, 2013, and January 31, 2021, inclusive (1L start date = index date). Patients also had documented HER2+ status, defined as any HER2+ (immunohistochemistry [IHC] 3+, IHC 2+/in situ hybridization+) test and the absence of a negative test (defined as IHC-negative 0-1+, fluorescence in situ hybridization negative/not amplified, negative not otherwise specified), on or before the index date, without discrepant results (eg, not having a record indicating HER2+ status and a record indicating HER2-negative status on the same day). Patients were required to have at least one record after the index date to minimize the chance of including patients who had moved to different care providers. They were excluded if they had used clinical trial drugs any time prior to or during first-line therapy, or if they had no treatment aside from hormonal therapy within the first 90 days after the mBC diagnosis date since patients with a 1L start later than clinically expected may reflect receipt of 1L treatment outside the Flatiron network. The data cutoff was January 31, 2022. Patients were followed longitudinally until death or last visit before data cutoff (data censoring). The baseline period was defined as all available data on or before the index date (start of 1L therapy).

### Baseline and treatment characteristics

Baseline demographic and clinical characteristics—including tumor status, disease characteristics, and g*BRCA* status—were extracted from the EHR. The type of BC treatment (agent or combination of agents) received in the first three LoTs (1L-3L) were categorized based on the 2021 NCCN guidelines.^18^

Flatiron’s existing LoT algorithm was slightly modified to reflect current clinical practice for patients with HER2+ mBC. Gaps in the exposure of up to 1 year were allowed within the same LoT, with the following constituting the start of a new LoT: the addition of a new HER2-directed drug or discontinuation of all HER2-directed drugs, changes in chemotherapy, or immunotherapy subclass, or the start of a new poly-adenosine diphosphate ribose polymerase inhibitor or a cyclin-dependent kinase 4/6 inhibitor, combined with hormonal therapy. All other changes to a regimen, including the addition of hormonal therapy alone, did not constitute a new LoT. Patients with HER2+ mBC were also required to have received treatment other than hormone therapy within 90 days of mBC diagnosis. Those patients with a longer gap prior to the first observed treatment may have received 1L therapy at a clinic outside Flatiron’s data network, leading to misclassification of a later LoT as 1L.

For injectable and infused therapies, the end date of treatment was defined as the date of last administration +21 days (the most common duration between administered treatments). The end date of oral medications was provided by Flatiron in the curated dataset. The end date of multidrug LoTs was the latest of these dates for the component agents.

### Outcomes

Each of the following outcomes was examined from the index date (start of 1L). Outcomes were also examined from the start of 2L, among patients with a second-line treatment.

#### Time to first subsequent therapy or death

Time to first subsequent therapy (TFST) was defined as the time from the start of the LoT to the start of the subsequent LoT or the date of death for patients who died without receiving a subsequent LoT. For patients with no indication of a further LoT or death, TFST was censored at the last activity date, defined as the last visit of any type during the study period.

#### Time to discontinuation or death

Time to discontinuation (TTD) was defined as the time from the start to the date of last administration +21 days for injectable and infused therapies or end date of oral medication or to the date of death for patients who died before the end of the LoT.

For patients still on treatment at the end of follow-up, patients with no indication of discontinuation of the current LoT or death were censored at their last activity date.

#### Real-world PFS

Real-world PFS (rwPFS) after the start of the LoT was calculated as the time from the start of the LoT to the first record of disease progression or death. Flatiron’s progression data defines an event where the treating clinician concluded that there was any growth or worsening in the disease of interest and is abstracted from unstructured data. Source evidence may be radiographic, pathologic, or from a physical exam. Some progression events are recorded in the Flatiron data as pseudo- or mixed-progression events; these were not considered true progression events and were excluded. For patients with no evidence of disease progression or death, rwPFS was censored at the last clinic note abstraction date, as progression events are mainly recorded in unstructured patient documents.

#### OS

OS for each patient was defined as the time from the start of the LoT to the date of death. For patients with no record of death, OS was censored at the last activity date during the study period. Month and year of death are noted in the Flatiron Health Data Repository; the day of death was imputed as the maximum of the date of the mid-point of the month of death or the last activity date across all medical records in that month. Patients who had more than one instance of a record that occurred after the month of death had their survival censored as of the latest date of a visit of any type, as it was unclear whether the patient had in fact died during the month on record.

### Statistical analysis

Baseline characteristics were summarized descriptively, using frequency counts and percentages for each category, with continuous variables reporting the median and range. The number of missing values was reported for each variable, and percentages for categorical variables were based on non-missing values. Kaplan-Meier methods were used for clinical outcome measures, which were reported from the start of both 1L and 2L.

Clinical outcomes were also assessed for subgroups of patients by HR status, 1L treatment type (THP vs other), and 2L treatment type (trastuzumab-based regimens vs T-DM1) based on the current treatment recommendations at the time.^9,18^ For analyses by 1L and 2L treatment type, weighted Kaplan-Meier plots were constructed, adjusting for baseline characteristics including age, race, practice type (academic or community), year of index date, BMI, stage at diagnosis, time since initial diagnosis, number and sites of metastases, presence of other primary cancer, HR status, and g*BRCA* status.

## Results

### Patient attrition

Of 28 161 patients with mBC in the Flatiron database, 22 201 (78.8%) were first diagnosed with mBC, and 19 165 (68.1%) received systemic anti-cancer therapy during the cohort identification period of January 2013 to January 2021. There were 3042 (10.8%) patients after excluding those without biomarker test results indicating HER2+ disease on or before the mBC diagnosis date (*n* = 16 105) and those with discrepant results on the same day (*n* = 18). Exclusion of patients<18 years of age (*n* = 0), without at least one day of follow-up after the index date (*n* = 18), use of investigational agents at any time before or during 1L treatment (*n* = 80), or no treatment besides hormonal therapy within 90 days after the mBC diagnosis date (*n* = 870) resulted in a sample size of 2074 (7.4%) patients in the final cohort.

### Baseline demographic and clinical characteristics

Of the 2074 patients with at least one LoT, the median age (range) was 61.0 (24-84) years. Most patients were white (62.7%), and 43.0% had stage IV disease at initial BC diagnosis. Approximately 63% and 34.7% had HR-positive (+) and HR-negative (-) disease prior to or at the start of 1L, respectively; 51 patients had unknown HR status (2.5%). The most common site of metastasis was bone (51.5%), followed by liver (33.2%) and lung (31.3%; Table 1).

**Table 1.** Baseline characteristics among patients with HER2+ mBC with ≥1 line of therapy^a^

	All patients with at least 1L *N* = 2074	Patients with HR+mBC and at least 1L *N* = 1303	Patients with HR− mBC and at least 1L *N* = 720
**Age (years) at start of 1L**			
Median (range)	61 (24-84)	61 (25-84)	60 (24-83)
**Age (years) at initial BC diagnosis**			
Median (range)	58 (23-83)	57 (23-83)	58 (24-83)
**Sex**			
Male	20 (1.0%)	18 (1.4%)	2 (0.3%)
Female	2054 (99.0%)	1285 (98.6%)	718 (99.7%)
**Race**			
White	1301 (62.7%)	826 (63.4%)	441 (61.3%)
Black or African American	246 (11.9%)	141 (10.8%)	100 (13.9%)
Asian	65 (3.1%)	42 (3.2%)	20 (2.8%)
Other	288 (13.9%)	179 (13.7%)	103 (14.3%)
Unknown	174 (8.4%)	115 (8.8%)	56 (7.8%)
**Time (days) from initial BC diagnosis to start of 1L**			
Number of observations	2073	1302	720
Mean (SD)	1058 (1,615)	1180 (1,746)	837 (1,290)
Median (range)	411 (0-15,520)	491 (1-15,520)	298 (0-10,720)
**Stage at initial breast cancer diagnosis**			
I	183 (8.8%)	109 (8.4%)	67 (9.3%)
II	435 (21.0%)	313 (24.0%)	116 (16.1%)
III	425 (20.5%)	248 (19.0%)	166 (23.1%)
IV	891 (43.0%)	546 (41.9%)	321 (44.6%)
Unknown	140 (6.8%)	87 (6.7%)	50 (6.9%)
**Number of metastatic sites at any time prior to or at start of 1L**			
1	867 (41.8%)	552 (42.4%)	290 (40.3%)
2	534 (25.7%)	333 (25.6%)	188 (26.1%)
3	306 (14.8%)	191 (14.7%)	109 (15.1%)
4+	212 (10.2%)	132 (10.1%)	75 (10.4%)
Unknown	155 (7.5%)	95 (7.3%)	58 (8.1%)
**Sites of metastases at any time prior to or at start of 1L**			
Bone	1068 (51.5%)	746 (57.3%)	297 (41.3%)
Brain	191 (9.2%)	96 (7.4%)	89 (12.4%)
Liver	689 (33.2%)	405 (31.1%)	266 (36.9%)
Lung	650 (31.3%)	382 (29.3%)	254 (35.3%)

Cell entries show *n* (%), unless otherwise specified.

^a^Fifteen one patients had missing HR status.

Abbreviations: 1L = first-line therapy; BC = breast cancer; HER2+ = human epidermal growth factor receptor 2 positive; HR = hormone receptor; HR- = hormone receptor negative; HR+ = hormone receptor positive; mBC = metastatic breast cancer; SD = standard deviation.

### Treatment characteristics by LoT

**Figure 1.**
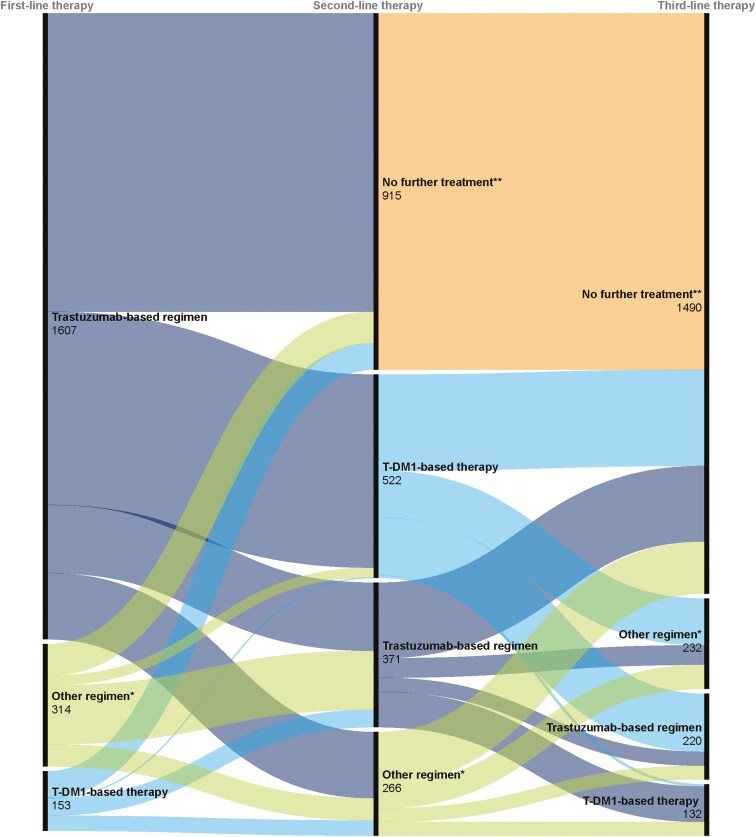
Treatment sequences from first- to third-line therapy in patients with HER2+metastatic breast cancer.

In the first LoT, 1,607 (77.5%) patients received a trastuzumab-based (T-based) regimen (Figure 1). The most common regimens across 1L included THP (38.9%), THP+ a platinum agent (7.5%), and T-DM1 monotherapy (6.1%).

The most common regimens across 2L (*n* = 1159) were T-DM1 monotherapy (35.7%), THP (11.2%) and T-DM1+ hormone therapy (8.0%). Among the 1607 patients who received any T-based regimen in 1L, 496 (30.9%) received a T-DM1-based regimen in 2L (Figure 1).

### Follow-up time and patient attrition between LoTs

Median (95% CI) follow-up time from the start of 1L was 26.0 months (12.6–44.7), and it was 18.1 months (7.8-13.1) from the start of 2L. After starting 1L (*n* = 2074), 55.9% of the patients received a subsequent LoT, while 18.1% of patients were still on 1L treatment, 20.2% had died, and 5.8% discontinued treatment without starting a new LoT at the end of the follow-up period.

After 2L therapy (*n* = 1159), 50.4% of patients received further treatment; at the end of the follow-up period, 17.9% were still on 2L treatment, while 25.9% and 5.9% had died and discontinued treatment, respectively.

### Clinical outcomes

**Figure 2.**
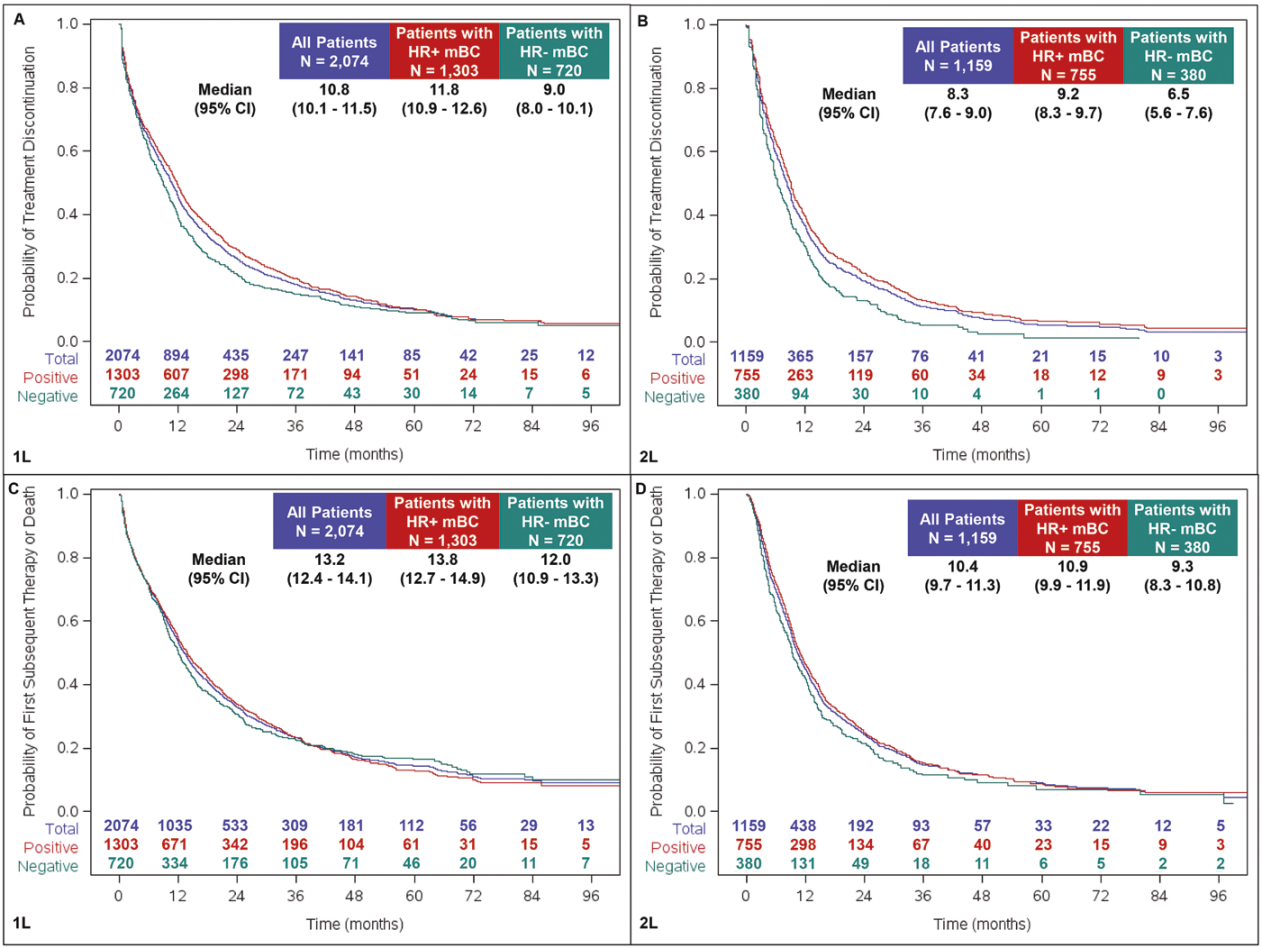

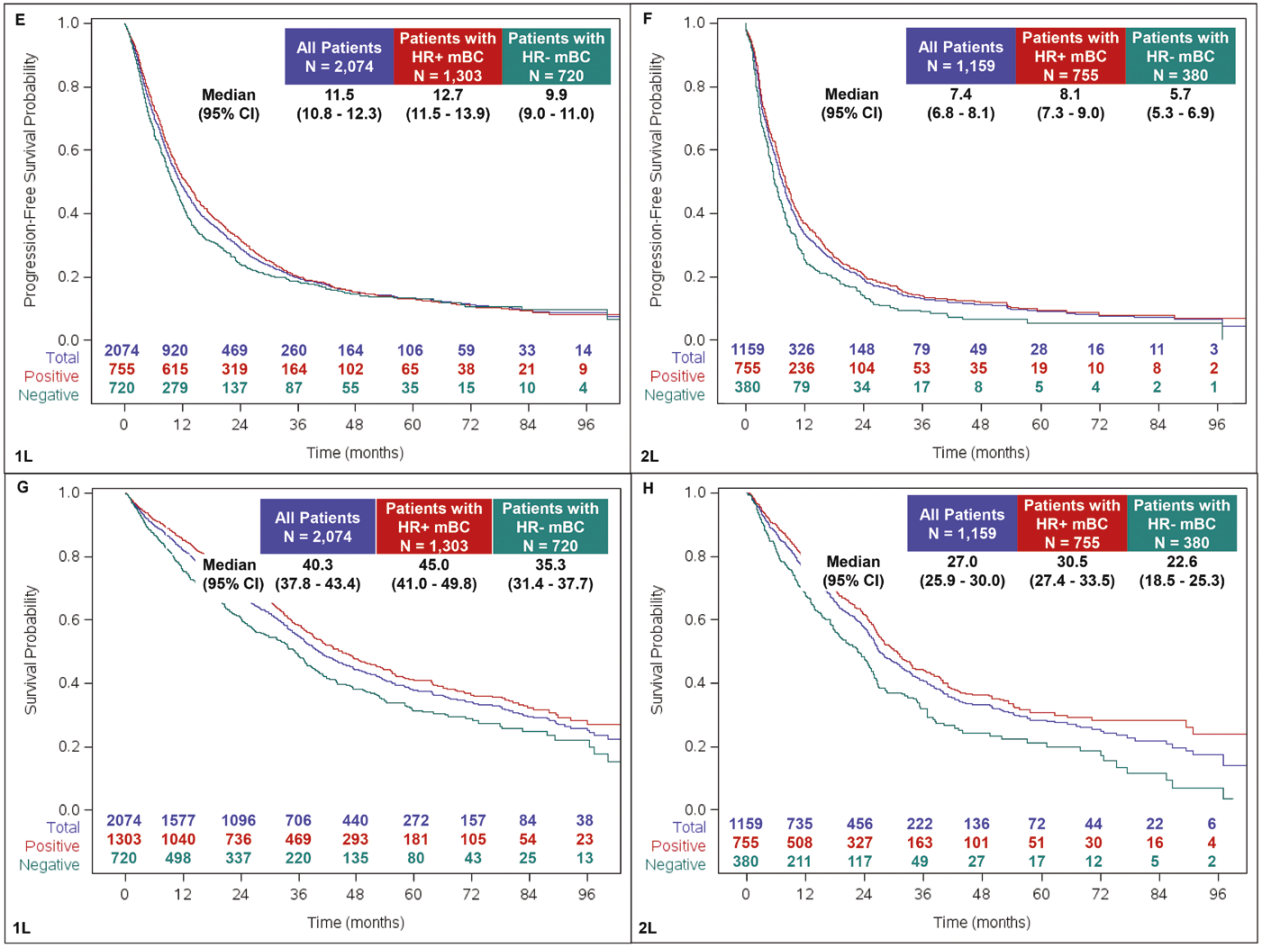
Time to treatment discontinuation (panels A, B), time to first subsequent therapy (C, D), real-world progression-free survival (E, F), and overall survival (G, H) following first- and second-line therapy.

From the start of 1L, overall median TTD was 10.8 months (95% CI, 10.1–11.5), median TFST was 13.2 months (95% CI, 12.4–14.1), and median rwPFS was 11.5 months (95% CI, 10.8–12.3). Median OS was 40.3 months (95% CI, 37.8–43.4; Figure 2).

Median TTD was 8.3 months (95% CI, 7.6-9.0) from the start of 2L, while median TFST was 10.4 months (9.7-11.3). From the start of 2L, the median rwPFS was 7.4 months (95% CI: 6.8–8.1) and median OS was 27 months (95% CI: 25.9-30.0; Figure 2).

Patients with HR+ mBC had an mOS of 45.0 (95% CI, 41.0-49.8) and 30.5 months (95% CI, 27.4-33.5) from the start of 1L and 2L, respectively; this was 35.3 (95% CI, 31.4-37.7) months from 1L and 22.6 months (95% CI, 18.5-25.3) from 2L for those with HR− mBC. Trends by HR status were similar for TTD, TFST, and rwPFS, with HR− mBC patients showing slightly shorter times to the respective event (Figure 2).

#### Supplementary material

TTD, TFST, rwPFS, and OS are presented for patients treated with THP and for those treated with other therapies in 1L (Figure S1). Patients treated with THP in 1L had longer clinical outcome times compared to patients treated with non-THP in 1L. Patients with HR+ mBC had longer-term outcomes compared to HR− mBC patients which was consistent across those with 1L THP and those with 1L non-THP. These outcomes are also presented for patients treated with trastuzumab-based regimens and those treated with T-DM1 in 2L (Supplementary Figure2). Patients with trastuzumab-based regimens in 2L had longer clinical outcomes than patients treated with T-DM1 in 2L. Patients with HR+ mBC had longer-term outcomes times compared to HR− mBC patients which was consistent across those with 2L trastuzumab-based regimens and those with 2L T-DM1.

## Discussion

Historically, SOC treatments for HER2+ mBC have been pertuzumab and trastuzumab combined with a taxane in 1L, and trastuzumab emtansine (T-DM-1) in the 2L. This study sought to examine alignment to clinical treatment guidelines, as well as patient attrition between LoTs. In the examination of 2074 patients treated for HER2+ mBC, 87.4% received anti-HER2-based therapy in 1L; however, there was considerable variability in the treatment selection with only 38.9% receiving THP and an additional 7.5% of patients receiving THP plus a platinum agent. In this 1L population, median rwPFS was 11.5 months (95% CI: 10.8-12.3) which is less than the 18 months mPFS seen in the clinical trial leading to approval of THP.^19^ After 1L, 55.9% (1159) of patients received a second LoT (18.1% remained on 1L treatment at data analysis). In patients who received 2L, only 35.7% received T-DM1 monotherapy and an additional 8.0% of patients received T-DM1+ hormone therapy. Median rwPFS was 7.4 months (95% CI, 6.8-8.1) in this 2L patient population, again potentially lower than previously expected from trials of the SOC.^20^ This retrospective real-world data shows that despite evidence-based clinical practice guideline recommendations, considerable variability is observed in treatment approaches for HER2+ mBC, which may impact outcomes.

In HER2+ mBC, though patients may see several LoTs, there is known attrition at each line of progression. In the follow-up of the 2074 patients on 1L therapy, 20.2% of patients died, and 5.8% discontinued treatment without starting a new LoT as of the end of the follow-up period. Of the 1159 patients on 2L therapy, 17.9% were still on 2L treatment, while 25.9% and 5.9% had died and discontinued treatment, respectively. Thus, just over 25% and 30% of patients died or discontinued therapy in 1L and 2L, respectively, without receiving further treatment for their HER2+ mBC. This highlights a need for more effective therapies that delay progression and provide longer clinical benefits in 1L and 2L HER2+ mBC. Two new treatments have been approved for patients who previously received at least one LoT including an anti-HER2-based therapy; T-DXd and tucatinib (in triplet combination) in 2019 and 2020, respectively.^15,16^ In May 2022, T-DXd was approved for use in 2L having previously been approved for third line and greater. These recently approved drugs may improve clinical outcomes for this population; however, patients who do not reach later LoTs are not eligible to receive these newly approved treatments. It will be important for future research to continue to evaluate the uptake and impact of these new drugs in the evolving treatment patterns landscape.

In this real-world study of 2074 patients treated for HER2+ mBC, 87.4% received anti-HER2-based therapy in 1L; however, this is higher than in a recent study utilizing data from the Tempus clinicogenomic US database, which found that among patients with HER2+ mBC, 74.2% received anti-HER2-based therapy in 1L.^21^ Unlike the current cohort, the Tempus study did not require treatment other than hormone therapy within the first 90 days after mBC diagnosis and found that 70% of those patients started their anti-HER2-based therapy within 3 months of their HER2 + test result.

In the current study, clinical outcomes differed by HR status. Patients with HR+ mBC had a median OS of 45.0 and 30.5 months from the start of 1L and 2L treatment, respectively. This duration was 35.3 months from the start of 1L treatment and 22.6 months from the start of 2L treatment for patients with HR- disease. Trends by HR status were similar for TTD, TFST, and rwPFS; these results are similar to those of other recent studies.^22-24^ The Systemic Therapies for HER2-Positive Metastatic Breast Cancer Study (SystHERs) was a prospective, observational disease registry that examined treatment patterns and clinical outcomes in patients with HER2 + mBC in real-world treatment settings, enrolled from June 2012 to June 2016.^25^ In SystHERs, patients with HR + disease had a longer median OS than those with HR- mBC (53.0 vs 43.4 months; hazard ratio 0.70; 95% CI, 0.56-0.87).^23^

### Strengths and llimitations

This study represents an examination of the treatment patterns and clinical outcomes of patients with HER2+ mBC. It was carried out using data recorded in a collection of electronic medical record systems that include clinical data such as biomarkers and staging. Our results are generalizable to a wide range of outpatient oncology practice groups in the United States, but may not represent all oncology practice sites within or outside the United States. As with any study using real-world data, the study is subject to limitations. There is a risk for misclassification bias, as some patients with HER2− mBC may have erroneously been classified as having HER2+ disease and included in the study cohort due to errors in test results or data entry into the EHR. Analyses stratified by treatment type used the weighted Kaplan-Meier approach in an effort to reduce selection bias, but no formal comparative analyses were conducted, and residual bias and confounding likely remain. As the Flatiron data is a site-based EHR database, numerous sources of information bias exist that cannot be mitigated. For example, it is possible that some patients who appeared to have discontinued their treatment may have moved to a different institution to continue their treatment while still having other encounters for a particular period of time after discontinuation, which would delay censoring based on visit dates. Information on surgery, radiation therapy, and other services received in hospitals was not available. Services and procedures provided outside of the Flatiron data network may not be documented in the database; however, the information in the data repository on treatments received in the oncology clinic and on outcomes such as OS is expected to be reliable, allowing a valid assessment of survival outcomes among patients with HER2+ mBC.

## Conclusion

Results from this real-world study of patients with HER2+ mBC receiving care in mainly community-based oncology clinics indicate that despite guideline recommendations, there is variability in treatment approaches for HER2 +  mBC, which may impact outcomes. Over half of the patients on 1L treatment had disease progression within 1 year of initiating treatment, and median overall survival from the start of 1L was just over 3 years. Approximately 25% of the patients died or discontinued their 1L therapy without receiving further treatment. This highlights a need for more effective therapies that delay progression and provide longer clinical benefits in earlier lines of therapy.

## Supplementary Material

oyae280_suppl_Supplementary_Figures_1

oyae280_suppl_Supplementary_Figures_2

## Data Availability

The data underlying this article will be shared on reasonable request to the corresponding author.
