# Supplementary figures and images for: Treatment patterns and associated outcomes among patients with HER2+ metastatic breast cancer in the United States: an observational cohort study
Source: Oncologist. 2025 Apr 7;30(4):oyae280. doi: 10.1093/oncolo/oyae280 (PMC11973896; doi:10.1093/oncolo/oyae280)

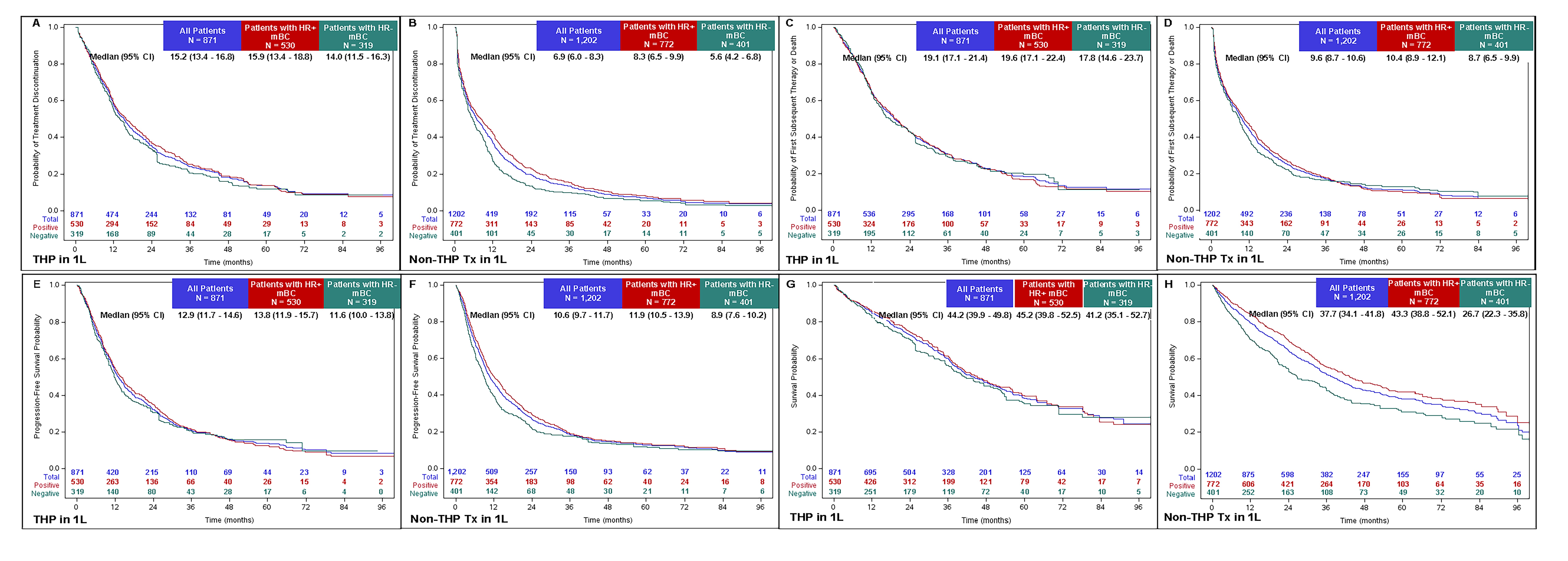

Supplement: oyae280_suppl_Supplementary_Figures_1 [file oyae280_suppl_supplementary_figures_1.jpeg]

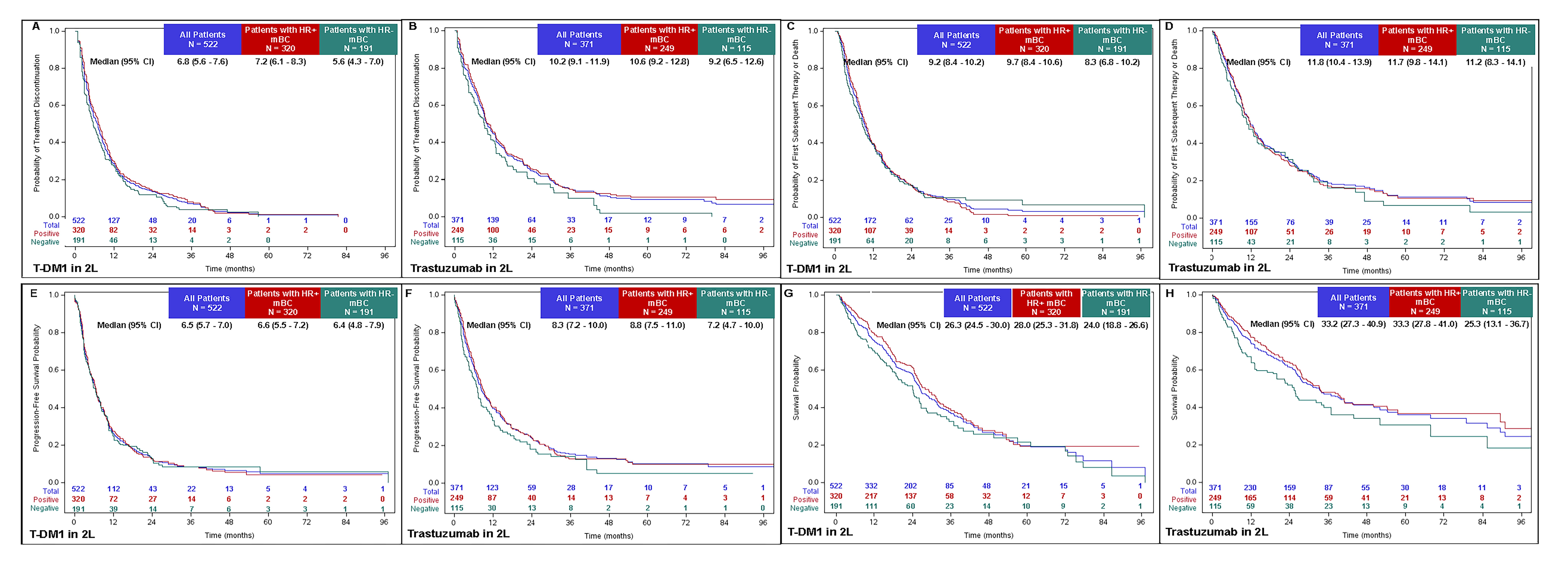

Supplement: oyae280_suppl_Supplementary_Figures_2 [file oyae280_suppl_supplementary_figures_2.jpeg]
